# Acute Thyroiditis in a Patient with Neck Trauma

**DOI:** 10.1155/2022/6126254

**Published:** 2022-12-05

**Authors:** Pearl Marian A. Peralta, Jeryl Ritzi T. Yu, Edrome F. Hernandez, Evangeline B. dela Fuente

**Affiliations:** ^1^University of the Philippines-Manila, Philippine General Hospital, Department of Psychiatry and Behavioral Medicine, Philippines; ^2^Center for Neurological Restoration, Neurological Institute, Cleveland Clinic, OH, USA; ^3^University of the Philippines-Manila, Philippine General Hospital, Department of Medicine, Philippines

## Abstract

The management of patients with major depressive disorder who present with self-injurious behavior is best optimized through a collaborative interprofessional approach. We describe a case of a 27-year-old woman without personal or family history of thyroid pathology who presented at the emergency department due to a suicide attempt by hanging. On examination, she was tachycardic with palpitations which persisted despite administration of analgesics and anxiolytics. Left temporal area swelling, left otorrhagia, and neck contusion were noted, involving consults with the Trauma, Neurosurgery, and Otorhinolaryngology teams. She was admitted to the psychiatric ward on account of persistent suicidal ideations. As part of the workup, thyroid function tests were done to rule out hypothyroidism as a cause of depressive symptoms. Results instead showed suppressed thyroid stimulating hormone and elevated free T4. Endocrinology service was consulted, and further workup showed absence of avid uptake of both thyroid glands on thyroid scan and undetectable thyrotropin receptor antibody level, supportive of a diagnosis of trauma-induced thyroiditis. This case increases awareness that trauma-induced thyroid dysfunction should be considered in patients with symptoms including, but not limited to, tachycardia and palpitations after a traumatic neck injury such as hanging.

## 1. Introduction

Suicide is recognized by the World Health Organization as a serious public health concern. Suicide mortality rate is an indicator of target 3.4 of the Sustainable Development Goals by 2030, which aims to reduce by one third premature mortality from noncommunicable diseases through prevention and treatment and promote mental health and well-being [1]. However, stigma continues to surround mental health disorders and suicide, which continue to impact the assessment and management of these conditions [2].

When a patient presents with self-injurious behavior associated with suicide, the condition is customarily labeled to be primarily a mental health disorder, and this may preclude the prudent consideration of any underlying or concomitant medical concerns prior to committing to a primarily psychiatric condition [3]. This is crucial especially among care providers in the emergency department—the first point of contact for patients who attempt suicide. Potential medical concerns, when not judiciously investigated, may be misattributed to psychiatric reasons and lead to a life-threatening medical crisis when not addressed appropriately.

We describe a case of a depressed young woman who attempted suicide by hanging and subsequently developed acute thyroid injury. Thyroid dysfunction may present with emotional and/or behavioral symptoms suggestive of a psychiatric symptomatology. These include agitation, anxiety, and insomnia which can be seen in a hyperthyroid state. It is not uncommon that a patient admitted in a psychiatric unit with these symptoms is mistakenly and readily viewed as purely psychiatric with no further assessment. Therefore, a high index of suspicion and a collaborative interprofessional approach are vital to guide appropriate management.

## 2. Case Presentation

A 27-year-old female without personal or family history of thyroid pathology presented at the emergency department (ED) due to a suicide attempt by hanging. Over the past two years, she had been experiencing feelings of inadequacy after discovering that her boyfriend was having an affair with another woman. Self-distraction techniques (immersion in work and spending time with her trusted friends) helped temporarily. However, early morning awakenings, difficulty concentrating, and feelings of worthlessness recurred, each episode lasting less than a week. Four months prior to the suicidal attempt, she demonstrated episodes of self-harm. Once, she stabbed herself in the right thigh after an argument as she was frustrated with her self-perceived worthlessness. She added that she wanted to be free from life's difficulties. One week prior, she became more socially withdrawn and refused to disclose her struggles or to seek professional help. She merely expressed “I want to rest” (translated from Tagalog/Filipino language). On the day of the attempt, the patient's mother found her hanging from approximately 6 feet high with her feet barely touching the floor. She cut the rope and the patient then fell to the ground, sustaining a head injury on the left temporal area.

**Figure 1:**
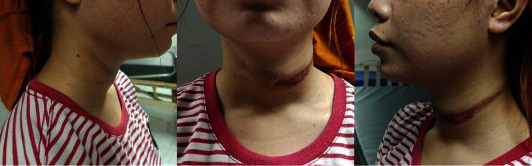
Neck contusion after suicide attempt by hanging.

She was seen in the emergency department awake with Glasgow Coma Scale 15, tachycardic at 104–120 bpm. There was no tremor or hypertension. The following findings were noted on physical exam: a neck contusion (Figure 1), a 5 × 5 cm hematoma on the left temporal region with dry blood outside the left external auditory canal, and a laceration on the left foot. Cranial, temporal bone, and cervical spine CT scan revealed an acute right-sided extra-axial and parenchymal hemorrhages with adjacent cerebral edema, extracalvarial soft tissue swelling or hematoma formation with irregularities, left fronto-temporo-parietal region, left temporal bone fracture with hemotympanum, and a straightened cervical spine. She was referred to Trauma, Neurosurgery, and Otorhinolaryngology services, and a nonsurgical management was deemed sufficient.

Due to persistent suicidal ideations, the patient was admitted to the psychiatric ward. She was started on escitalopram 10 mg/tab, 1/2 tab once a day then adjusted to 1 tab once a day and a low-dose aripiprazole 5 mg/tab, and 1/2 tab before bedtime to address her depressive symptoms and reduce her impulsivity of self-harm, respectively. As part of the workup, a thyroid function test was done to rule out a primary medical condition such as hypothyroidism, which may manifest as depressive symptoms. Results instead were supportive of a hyperthyroid state. Thyroid stimulating hormone (TSH) was suppressed at 0.1584 uIU/mL (range: 0.35-4.94); free T4 (FT4) was slightly elevated at 19.27 pmol/L (range: 9.01-19.05), and free T3 was normal at 4.22 pmol/L (range: 2.89-4.88). These were found to be congruent with the physical exam finding of tachycardia and palpitations which did not abate despite anxiolytics as well as analgesics for headache and neck pain from the fall. There was no history of intake of psychoactive substances. She denied any history or symptoms of previous thyroid disease such as dyspnea, palpitations, tremor, weight loss, goiter, exophthalmos, or recent viral infection. There was no evidence of enlarged thyroid gland and other signs of hyperthyroidism on physical exam. Pregnancy test was negative.

**Figure 2:**
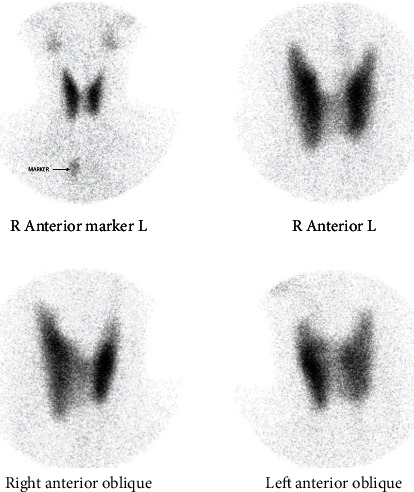
Tc-99 m pertechnetate thyroid scintigraphy (thyroid scan) showing absence of avid uptake of both thyroid glands with no ectopic thyroid tissue and no scarring.

Endocrinology service consult was requested for further evaluation of thyroid function. Thyroid scan (Tc 99 scintigraphy) done on the 6th day post neck trauma showed absence of avid uptake of both thyroid glands with no ectopic thyroid tissue and no scarring (Figure 2). Thyrotropin receptor antibody (Trab) level was undetectable 0 u/L (<1 Negative). Given the clinical course along with the thyroid scan and Trab results, the impression was trauma-induced thyroiditis, which did not warrant initiation of antithyroid drugs. Propranolol was ordered on as needed basis for symptomatic control, with spontaneous resolution of tachycardia and palpitations. The patient was discharged stable. Supportive psychotherapy and psychoeducation were continued, and she had significant mood improvement and reduced impulsivity to self-harm. Four weeks after discharge, the patient returned to work with improved mood, social skills, and relationships.

## 3. Discussion

While suicide is preventable, over 700,000 people die due to suicide every year. Over 77% of suicides occurred in low and middle-resource countries in 2019 [1]. In the Philippines, the most recent crude suicide rates per 100,000 population was 4.58 males per 100,000 population ages 25-34 years and 1.41 females per 100,000 population in the same age group since July 2021 [1]. Among Filipino youth, ages 15 to 27 years, the methods of suicide were classified as either violent or nonviolent. Violent methods include slashing of wrists, stabbing oneself, hanging, throwing oneself under a vehicle, and shooting oneself with a gun, while nonviolent methods involve substance ingestion and starving oneself [4]. Hanging, shooting, and organophosphate ingestion were the most common reported methods of suicide [5].

We presented a case of a depressed young woman who attempted suicide by hanging and subsequently developed acute thyroiditis. Although infection, surgery, and withdrawal of medications are the most common precipitating events of hyperthyroidism and thyroid storm, trauma may also result in thyroid abnormalities. Derangements in thyroid hormone levels have been detected immediately following traumatic injury [6, 7], and the general pattern involves a triphasic course which starts with a temporary thyrotoxicosis that proceeds to hypothyroidism, followed by recovery [8].

The mechanism and predictors of thyroid abnormalities precipitated by blunt neck trauma continue to be explored. Thyroid abnormalities postmanipulation have been described as early as 1975 when nonspecific multifocal granulomatous folliculitis was documented in thyroid glands removed postsurgery [9]. This was named “palpation thyroiditis” to denote effects from palpation of the glands during surgery. Subsequent reports of thyroid abnormalities following thyroid-related surgery were reported [10, 11]. One hypothesis explaining changes in thyroid hormone levels after direct trauma to the thyroid gland is hormone release from the ruptured acini due to local injury, resulting in a hyperthyroid state [12]. In a postmortem analysis, FT3 and thyroglobulin levels were significantly higher in cases of hanging compared to cases of sudden death [13]. Trauma to sites that were remote from the thyroid gland has also resulted in thyroid storm, and the risk is further increased when there is previous history of hyperthyroidism [12, 14].

Among cases of trauma localized to the thyroid gland, there are known cases of thyrotoxicosis and thyroid storm induced by strangulation in patients with undiagnosed Graves' disease [15, 16]. Limited reports of thyroid abnormalities after hanging and strangulation have been documented in patients without underlying thyroid disease. Thyroid hormone level changes were detected as early as hours after hanging and strangulation [17, 18] and as late as eight weeks after athletic injury [19]. One report described a 19-year-old woman without history of thyroid disease, who was admitted following a suicide attempt by hanging that was complicated by postasphyxiation from cardiac arrest [17]. She was revived after cardiopulmonary resuscitation, however, remained to be tachycardic, unresponsive, and hypertensive. Initial biochemical tests showed suppressed TSH < 0.01 mIU/ml (range: 0.2-4.0) and normal free T4 and T3. On monitoring, trends of FT4 and FT3 gradually increased and doubled in two weeks. She was managed as thyroid storm and symptoms resolved on initiation of methimazole and beta blockers. Another case was a 37-year-old woman without history of thyroid disease who was found with a tourniquet tied around her neck secondary to an assault [18]. In the ED, she was seen tachycardic and febrile with decrease in sensorium and cervical contusion. Despite appropriate fluid administration and antipyretics, her condition deteriorated and eventually led to multiple organ dysfunction. Thyroid function tests showed suppressed TSH 0.01 mU/L (range: 0.4-5.0) and elevated free T4 19.3 ug/dL (range: 5-12). Treatment for thyroid storm was administered resulting in dramatic improvement of symptoms.

Thyroid hormone abnormalities can also present subacutely. A 15-year-old man experienced palpitations, weight loss, asthenia, fatigue, diaphoresis, and tremor for eight weeks after an episode of hanging during a judo competition [19]. Workup showed low TSH and elevated free T4. Autoimmune studies (anti-peroxidase and anti-thyroglobulin antibodies) were negative. Thyroid scan (Tc 99 scintigraphy) showed a low uptake, and he was managed as a case of subacute thyroiditis posttrauma. Beta-blocker was initiated, and a repeat thyroid function test three months later showed euthyroid state. Our patient presented with normal uptake of both thyroid glands on thyroid scan six days postinjury. In the event that she presents with recurrence of symptoms weeks to months postinjury, it will be worthwhile to reevaluate for delayed posttraumatic thyroid pathology.

Thyroid complications post neck trauma represent a spectrum, ranging from thyroiditis as seen in our case to thyroid storm. Our patient did not have known history of thyroid disease or physical findings that would support hyperthyroid states such as Graves' disease prior to the attempted suicide. Furthermore, workup demonstrated a normal thyroid scan. This makes it less likely that the hyperthyroid state was secondary to a thyroid pathology prior to neck trauma. A hyperthyroid state secondary to an autoimmune condition such as Grave's disease is also unlikely given the undetectable thyrotropin receptor antibody level [8]. Considering the clinical and diagnostic parameters, her manifestations are less likely to be attributed to possible unknown preexisting thyroid pathology. To further support the patient's thyroid pathology as a sequelae of neck trauma, a repeat thyroid function test may be considered once fully recovered to document euthyroid state.

In summary, we presented a case of a depressed young woman who suffered from neck trauma secondary to attempted suicide by hanging and subsequently developed acute thyroiditis. A patient who presents with a suicide attempt must first be stabilized (securing the airway, maintaining ventilation, controlling hemorrhage, and treating shock) [20]. It is prudent to rule out intoxication and delirium, along with potential medical causes of the patient's depressed state. Furthermore, a careful evaluation of symptoms and signs is warranted to diagnose thyroid dysfunction among patients experiencing traumatic neck injury from hanging or strangulation. Although thyroid function test is not customarily included in the workup of patients with blunt neck injury, symptoms such as persistent tachycardia and palpitations despite administration of anxiolytics should prompt a high index of suspicion and evaluation for possible thyroid dysfunction secondary to neck trauma. Timely assessment and diagnosis will inform appropriate management and prevent any potential untoward sequelae.

The learning points from this case are as follows:

1. The management of patients with attempted suicide involves not only psychiatry but also other services depending on the patient's method of suicide and clinical presentation. A collaborative interprofessional approach is strongly recommended to better assess and manage patients who present with self-injurious behavior. This prevents symptom exacerbation and avoids possible life-threatening sequelae.
2. Thyroid dysfunction can occur among patients who present with blunt neck trauma even without a preexisting personal or family history of thyroid disease. A high index of suspicion for trauma-induced acute thyroiditis is strongly recommended among patients with blunt neck trauma due to a suicide attempt by hanging especially those who present with anxiety, palpitations, and persistent tachycardia.
3. We urge healthcare providers to practice proper attribution of symptoms secondary to thyroid dysfunction versus misattribution solely to a psychiatric diagnosis to better understand symptoms and inform appropriate management.
